# Screening and identification of tissue-infiltrating immune cells and genes for patients with emphysema phenotype of COPD

**DOI:** 10.3389/fimmu.2022.967357

**Published:** 2022-09-27

**Authors:** Di Wang, Bingnan Chen, Shuang Bai, Li Zhao

**Affiliations:** ^1^ Department of Pulmonary and Critical Care Medicine, Shengjing Hospital of China Medical University, Shenyang, China; ^2^ Department of Obstetrics and Gynaecology, The First Affiliated Hospital of Chongqing Medical University, Chongqing, China

**Keywords:** emphysema phenotype, tissue-infiltrating immune cells, SERPINA3, emphysema risk prediction model, bioinformatics

## Abstract

**Objective:**

To study the tissue-infiltrating immune cells of the emphysema phenotype of chronic obstructive pulmonary disease (COPD) and find the molecular mechanism related to the development of emphysema to offer potential targets for more precise treatment of patients with COPD.

**Methods:**

Combined analyses of COPD emphysema phenotype lung tissue-related datasets, GSE47460 and GSE1122, were performed. CIBERSORT was used to assess the distribution of tissue-infiltrating immune cells. Weighted gene co-expression network analysis (WGCNA) was used to select immune key genes closely related to clinical features. Rt-qPCR experiments were used for the validation of key genes. Emphysema risk prediction models were constructed by logistic regression analysis and a nomogram was developed.

**Results:**

In this study, three immune cells significantly associated with clinical features of emphysema (FEV1 post-bronchodilator % predicted, GOLD Stage, and DLCO) were found. The proportion of neutrophils (p=0.025) infiltrating in the emphysema phenotype was significantly increased compared with the non-emphysema phenotype, while the proportions of M2 macrophages (p=0.004) and resting mast cells (p=0.01) were significantly decreased. Five immune-related differentially expressed genes (DEGs) were found. WGCNA and clinical lung tissue validation of patients with emphysema phenotype were performed to further screen immune-related genes closely related to clinical features. A key gene (SERPINA3) was selected and included in the emphysema risk prediction model. Compared with the traditional clinical prediction model (AUC=0.923), the combined prediction model, including SERPINA3 and resting mast cells (AUC=0.941), had better discrimination power and higher net benefit.

**Conclusion:**

This study comprehensively analyzed the tissue-infiltrating immune cells significantly associated with emphysema phenotype, including M2 macrophages, neutrophils, and resting mast cells, and identified SERPINA3 as a key immune-related gene.

## Introduction

Chronic obstructive pulmonary disease (COPD) is a group of lung diseases characterized by airflow limitation. The medical and social pressures caused by COPD are increasing with the gradual growth in the aging population. As of June 2021, COPD was recognized by the World Health Organization as the third leading cause of death globally, causing 3.23 million deaths in 2019 (1). Many studies suggest that COPD is highly heterogeneous, with etiologies ranging from small airway lesions to alveolar parenchyma destruction that can lead to COPD. Therefore, COPD appears to be a syndrome rather than a single disease (2, 3). COPD is subdivided into different phenotypes to understand the course of disease development better and find more targeted treatments. The emphysema phenotype is an independent phenotype mainly manifested by the destruction of respiratory bronchiolar walls and alveoli, followed by excessive expansion and inflation of lung tissue and weakened lung elasticity (4). This phenotype has more severe dyspnea symptoms and poorer exercise tolerance, with higher mortality and more serious complications than other phenotypes (5). Many smokers with obvious emphysema and lung tissue damage show preserved pulmonary function (6, 7). These smokers should receive early intervention to prevent the progression of structural destruction and improve their quality of life (8). As a result, it is crucial to examine the pathogenesis of the emphysema phenotype of COPD and identify potential targets.

In the emphysema phenotype of COPD, many aspects of the innate and adaptive immune responses are abnormal (9). Neutrophils, which are significantly increased in sputum and blood in patients with emphysema, release elastase that breaks down the extracellular matrix, destroying lung tissue (10). Decreased phagocytic activity of macrophages increases the persistence of the inflammatory process (11), and persistent chronic stimulation may affect T cell numbers (12). These abnormalities may result in antigen-specific immune responses, leading to repeated exacerbations and infections in the late stages of COPD (13, 14). Smoke exposure also leads to innate immune responses and the release of some cytokines, such as CXCL8, IL-6, TNF, and leukotriene B4, which can induce the infiltration of neutrophils and monocytes. These inflammatory cells can activate oxidative stress and protease-antiprotease imbalance, causing epithelial damage and cell death (15). Thus, changes in the lung immune microenvironment are closely related to the development of the emphysema phenotype.

However, previous studies on immune cell infiltration in the emphysema phenotype of COPD have focused on a single sample or single immune cell using immunohistochemical staining or flow cytometry (16, 17), which cannot comprehensively assess the overall states of immune infiltration of emphysema. We selected the lung tissue-related data of patients with emphysema phenotype in the Gene Expression Omnibus (GEO) database. The infiltration of immune cells in lung tissue was assessed by bioinformatics methods to identify key immune cells related to emphysema. Further analyses were performed by looking for differentially expressed genes and using a weighted co-expression network analysis (WGCNA) to screen out key immune-related genes. We aimed to achieve this by better mapping the role of immune cells in the pathogenesis of emphysema and by identifying risk factors for emphysema through important immune cells and genes.

## Material and methods

### Data preparation and download

The datasets GSE47460 and GSE1122 were selected and downloaded from the public database Gene Expression Omnibus (GEO, http://www.ncbi.nlm.nih.gov/geo). Combined analysis was performed to screen out key genes more precisely. GSE47460 is multi-batch microarray dataset which contains 2 platforms, GPL6480 and GPL14550. In order to avoid batch differences, 10% of the samples were randomly selected for repetition in each platform. Emphysema patients in this dataset were determined based on the CT-emphysema index. Patients with -950 HU value≥15% were diagnosed with emphysema (18). The detailed information of the selected datasets was shown in **Table 1**. The GSE1122 dataset contains 15 samples. We only included 5 emphysema samples and 5 non-emphysema samples, and the other 5 samples with antitrypsin deficiency were not included in this study. The gender and age of the patients included in the GSE47460 and GSE1122 datasets were shown in **Supplementary Table 1**, and there was no significant difference between the datasets.

**Table 1.** Clinical characteristics of 220 samples in GSE47460 dataset.

Characteristics	emphysema(n=78)	control(n=142)	P value
FEV1(%)	36.28±17.51	66.89±16.01	1.62342E-19
FVC(%)	71.89±19.03	87.54±13.56	1.13407E-06
DLCO(%)	37±13.72	66.41±19.89	4.23925E-21

### Analyses of immune infiltration in the lung tissue with emphysema

CIBERSORT is a method to characterize the composition of immune cells in the microenvironment from gene expression profiles data (19). We downloaded the CIBERSORT R source code and LM22 gene set from the CIBERSORT website (http://cibersort.stanford.edu/) and ran it locally. The expression profiles of the GSE47460 dataset in the GPL6480 and GPL14550 platforms were normalized using R software. The abundance matrix of 22 immune cells was obtained from the gene expression profile and visualized using the “graphics”, “pheatmap” and “vioplot” packages in R. Next, spearman correlation analysis was performed using the “corplot” package in R on the normalized GPL6480 and GPL14550 platform data to analyze the relationship between immune cell abundance and clinical characteristics of emphysema patients, including forced expiratory volume in the first second (FEV1), forced vital capacity (FVC), GOLD Stage and diffusion lung capacity for CO (DLCO).

### Immune-related differentially expressed genes screening

Analysis of differentially expressed genes in GSE1122 dataset and GSE47460 dataset were carried out using GEO2R and the intersection of results was taken. GO (Gene Ontology) and KEGG (Kyoto Encyclopedia of Genes and Genomes) enrichment analyses were performed using the metascape website tool (https://metascape.org/gp/index.html), and the top 20 pathways were displayed. The genes enriched in immune-related pathways were selected as immune-related differentially expressed genes (DEGs) in emphysema patients.

### Weighted gene co-expression network analysis

In order to further screen out the genes closely related to clinical characteristics from the above immune-related DEGs, we extracted a total of 15180 genes from the expression matrix of the GSE47460 dataset in the GPL6480 and GPL14550 platforms using the “WGCNA” package in R for the construction of co-expression networks. In this study, we chose a soft threshold β = 5 (scale-free R2 = 0.8743). Subsequently, we transformed the adjacency matrix into a topological overlap matrix and merged similar modules after a height cutoff of 0.85. Next, we calculated pearson correlation coefficients between modules and clinical data to select key modules closely related to clinical features of emphysema.

### Determination of key genes and Rt-qPCR experiments

The above immune-related DEGs were intersected with the genes in the key modules selected by WGCNA to obtain DEGs related to both immune and clinical features. Next, in order to screen out key genes more precisely and to further verify the level of tissue-infiltrating immune cells, lung tissues were collected from 5 patients with emphysema and 5 patients without emphysema. All samples in this study were obtained from patients with lung tumor. The sampling location is 5 cm next to the tumor All samples were obtained under the informed consent of patients. Total RNA from each sample was extracted using RNAiso Plus (TaKaRa, Beijing, China). Reverse transcription was performed using PrimeScript™ RT reagent Kit with gDNA Eraser (TaKaRa, Beijing, China). TB Green^®^ Premix Ex Taq™ (Tli RNaseH Plus)(TaKaRa, Beijing, China) was used to quantitatively detect the mRNA levels of key genes, in which GAPDH gene was used as an endogenous control for mRNA normalization. Primer sequences for each gene are shown in **Supplementary Table 2**. Comparisons were analysed by t-test. To further clarify the association between key genes and tissue-infiltrating immune cells, spearman correlation analysis was performed between the expression levels of key genes and the levels of lung tissue-infiltrating immune cells in the GSE47460 dataset.

### Construction of emphysema risk prediction model

The hub genes, immune cells and clinical features obtained in the above analysis were included in the univariate analysis. The t-test or Mann-Whitney U test was used for continuous variables, while the χ2 test or Fisher’s exact test was used for categorical variables. Factors with P < 0.2 in the univariate analysis were included in multivariate logistic regression analysis, in which forward stepwise algorithms and likelihood ratio tests were used. Two emphysema risk prediction models were constructed, including a clinical prediction model (clinical features only) and a combined prediction model (hub genes, immune cells and clinical features). Hosmer-Lemeshow test (H-L test) and calibration curve were used to evaluate the calibration power of models, while the receiver operating characteristic (ROC) curve and the area under the ROC curve (AUC) were used to evaluate the discrimination power of models. Finally, a nomogram was developed through the “rms” package in R to visualize the prediction model.

### Western blot analysis

Western blotting was performed using lung tissues collected from 4 patients with emphysema and 4 patients without emphysema. Inclusion criteria for all samples were the same as those for Rt-qPCR. Proteins were extracted using RIPA lysis buffer, and the concentration of protein was quantified using the BCA protein concentration assay kit (Solarbio, China). Equal amounts of denatured proteins were loaded in sodium dodecyl sulfate–polyacrylamide gel electrophoresis and transferred to a polyvinylidene fluoride membrane. The membrane was blocked with 5% non-fat milk for 2h, and incubated overnight with the primary antibody at 4°C. The following antibodies were used: anti-GAPDH and anti-SEPRINA3 from Proteintech (Chicago, IL, United States). On the following day, the membranes were washed in TBST (10 mM Tris-HCl pH 7.4, 100 mM NaCl, 0.5% Tween-20) and incubated with secondary antibodies for 1.5 h. The membranes were visualized using enhanced chemiluminescence reagent for chemiluminescence detection. ImageJ software was used for analyzing final images.

All analyses in this study were performed in SPSS software (version 25.0, IBM Corporation, Armonk, New York), STATA software (version 17, Stata Corporation, Calgary, Texas) and R language (version 4.1.2, R core development team).

## Results

### Immune landscape of patients with COPD emphysema phenotype

**Figure 1:**
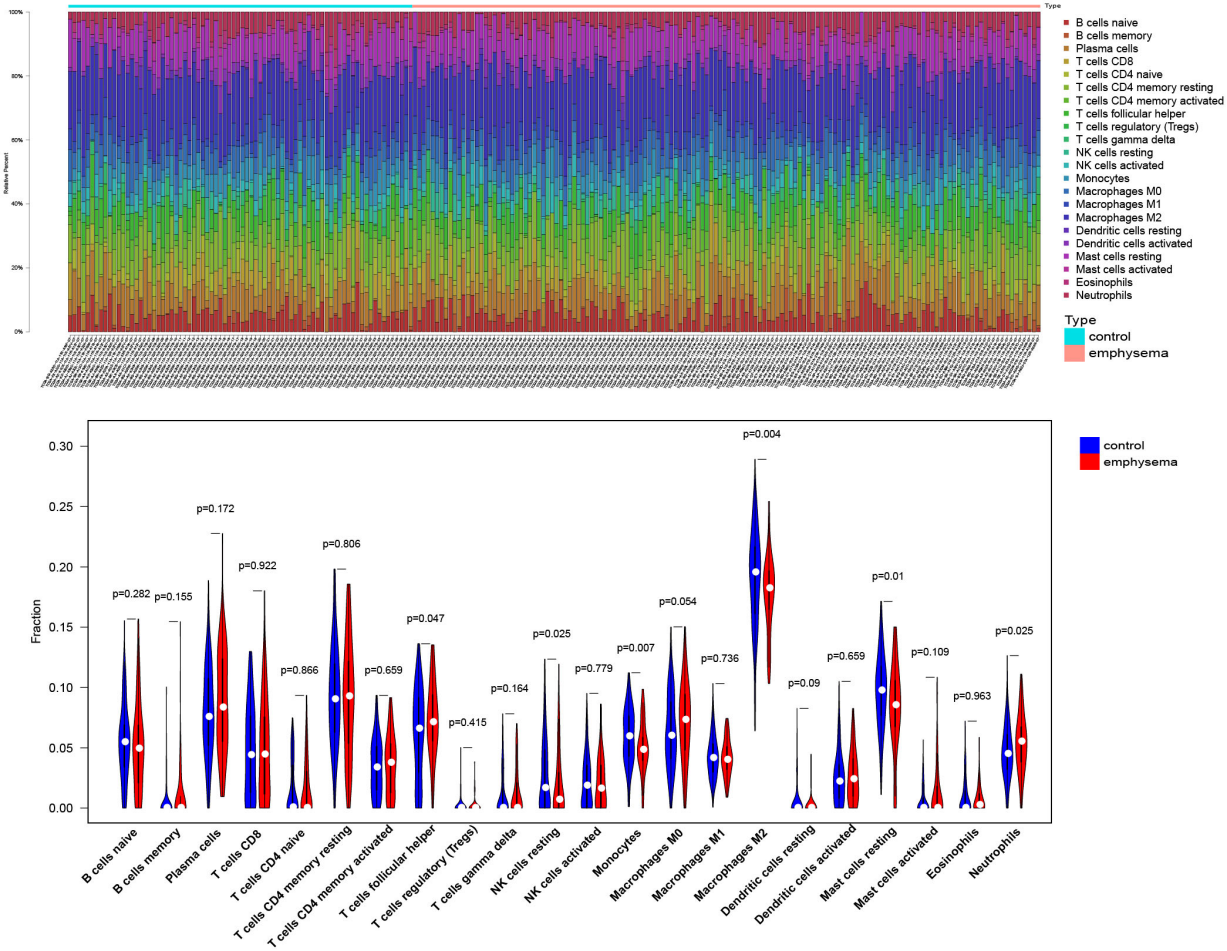
The landscape of lung tissue-infiltrating immune cells. Bar plot **(A)** and violin plot **(B)** shows the distribution of 22 types of immune cells in emphysema phenotype patients and non-emphysema phenotype patients with COPD.

**Figure 2:**
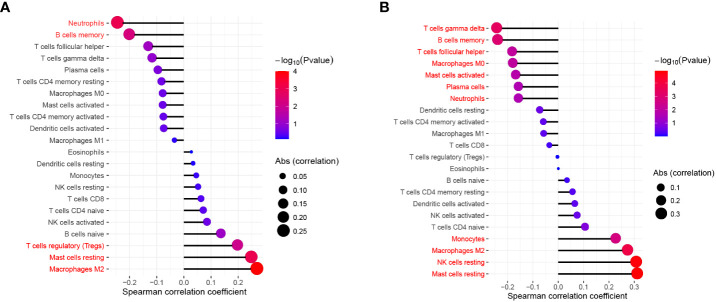
The relationship between FEV1 post-bronchodilator % predicted **(A)**, DLCO **(B)** and the infiltration level of lung tissue-infiltrating immune cells; Statistical significance (P<0.05) in red font.

**Figure 3:**
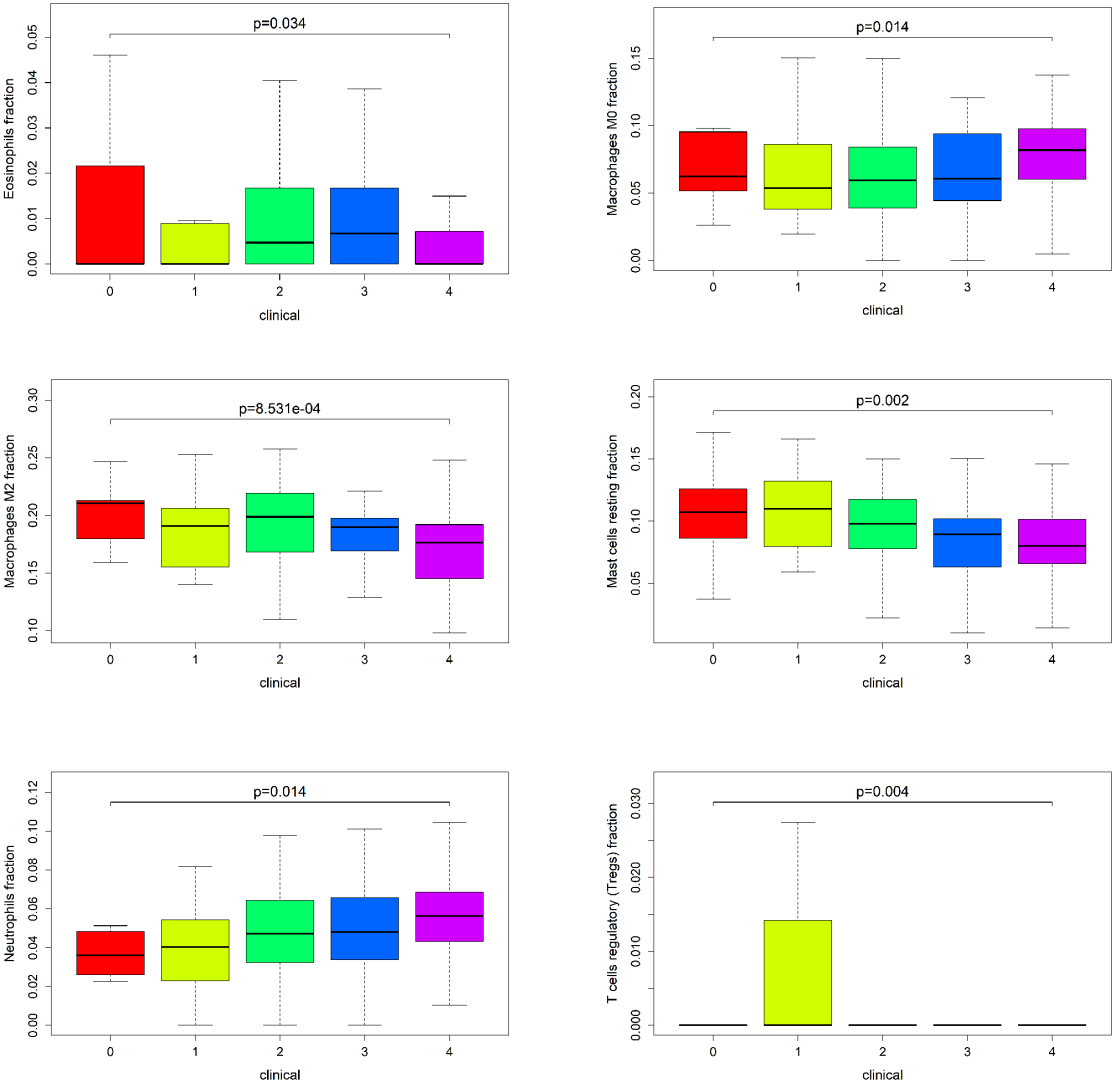
The relationship between GOLD Stage and the infiltration level of statistically significant (P < 0.05) tissue-infiltrating immune cells.

To explore the immune landscape of patients with emphysema and those without emphysema, we analyzed the expression matrix in the GSE47460 dataset, which contains a total of 220 lung tissues. Differences in immune infiltration of 78 patients with emphysema and 142 patients without emphysema were analyzed by CIBERSORT. The abundance ratio matrix of 22 immune cells in 220 lung tissues is shown in **Figure 1A** (**Supplementary Table 3**). According to the violin plot in **Figure 1B**, the proportions of T cells follicular helper (p=0.047) and neutrophils (p=0.025) in lung tissue of patients with emphysema were significantly higher than those without emphysema, while the proportions of NK cells resting (p=0.025), monocytes (p=0.007), macrophages M2 (p=0.004), mast cells resting (p=0.01) were relatively low. We used the Wilcoxon signed-rank-sum test to analyze the correlation between immune cell abundance and emphysema-related clinical features (FEV1 post-bronchodilator % predicted, GOLD Stage, and DLCO) with p<0.05 as the critical value. We found that many immune cells were associated with them, albeit weakly. The results are shown in **Figures 2**, **3**. Three immune cells, including M2 macrophages, neutrophils, and resting mast cells, were significantly associated with the clinical features of emphysema.

### Identification of differentially expressed genes and enrichment analysis

**Figure 4:**
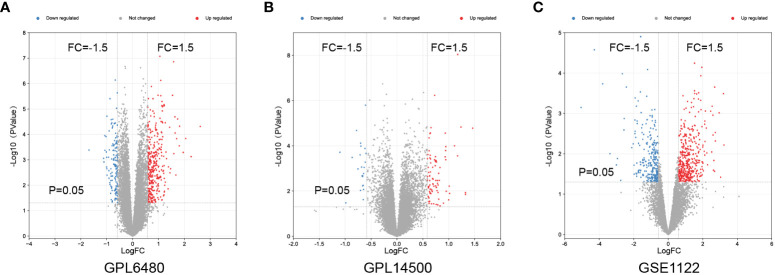
Differential expressed genes analysis. Volcano maps of GPL6480 **(A),** GPL14500 **(B)** in GSE47460 and GSE1122 **(C)**.

**Figure 5:**
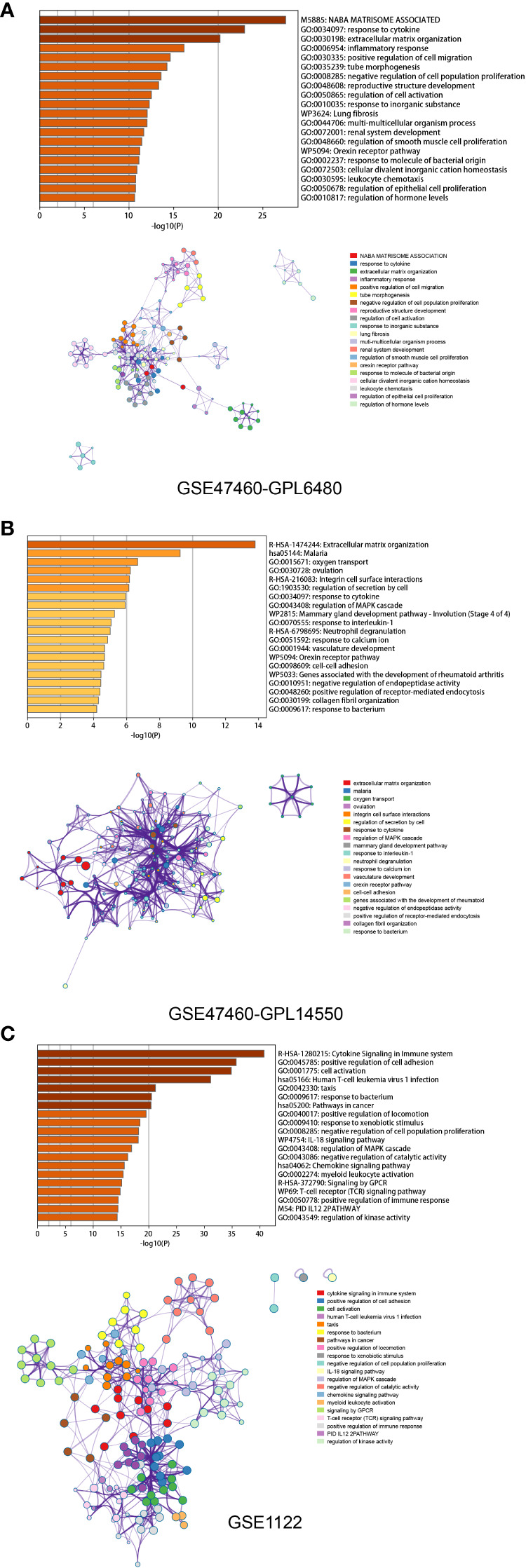
Functional enrichment analysis based on Metascape of GPL6480 **(A),** GPL14500 **(B)** in GSE47460 and GSE1122 **(C)**. Bar graph demonstrates P-value of enriched clusters. Each node in network represents an enriched term of corresponding clusters. For large clusters, the network only displays the top 10 terms with P-value. The edge reflects the connection of functions and pathways. The size of the nodes reflects the number of genes, and the thickness of the edges reflects correlation level of terms.

To identify differentially expressed genes (DEGs) associated with the emphysema phenotype, we found 586, 479, and 109 DEGs (p<0.05 and |fold change|>1.5) in the GSE1122 dataset and two platforms of GSE47460 dataset (GPL6480 and GPL14550), respectively. The results are shown in volcano plots (**Figures 4A–C**). Functional enrichment analysis of DEGs was performed, and multiple immune function-related pathways were enriched in both datasets, such as cytokine signaling in the immune system, human T-cell leukemia virus infection, response to the bacterium, response to cytokine, and inflammatory response (**Figures 5A–C**). To further screen immune-related DEGs in patients with emphysema, we sorted out the DEGs in immune-related pathways, took the intersection of DEGs in these pathways, and finally obtained five immune-related key genes.

### Identification of key gene modules by WGCNA

**Figure 6:**
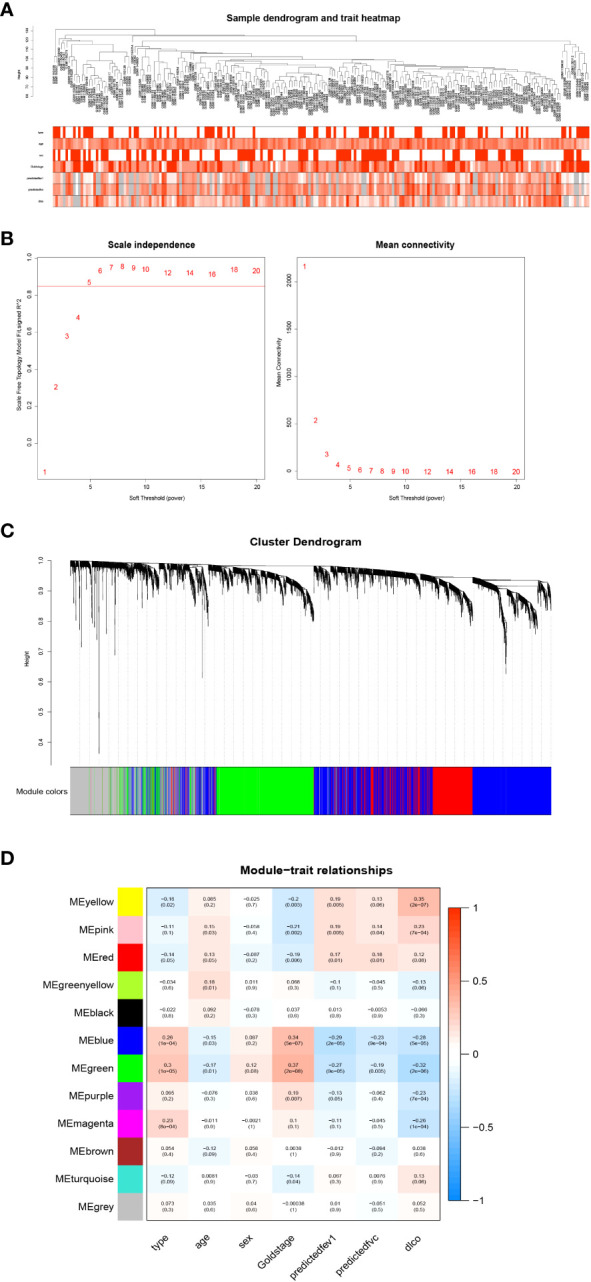
Weighted co-expression network analysis. **(A)** Sample dendrogram and trait heatmap; **(B)** Analysis of the scale-free fit index (left) and the mean connectivity (right) for various soft-thresholding powers; **(C)** Clustering dendrograms of genes based on a dissimilarity measure; **(D)** Module-trait associations were evaluated by correlations between module eigengenes and sample traits.

**Figure 7:**
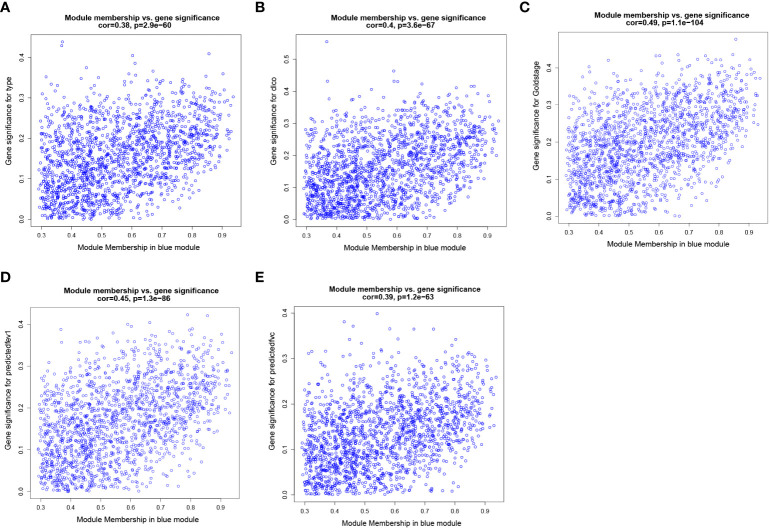
Weighted co-expression network analysis. Scatter plot of genetic significance of emphysema phenotype **(A)**, DLCO **(B)**, GOLD Stage **(C)**, FEV1 post-bronchodilator % predicted **(D)** and FVC post-bronchodilator % predicted **(E)** *vs*. Module Membership in blue module.

**Figure 8:**
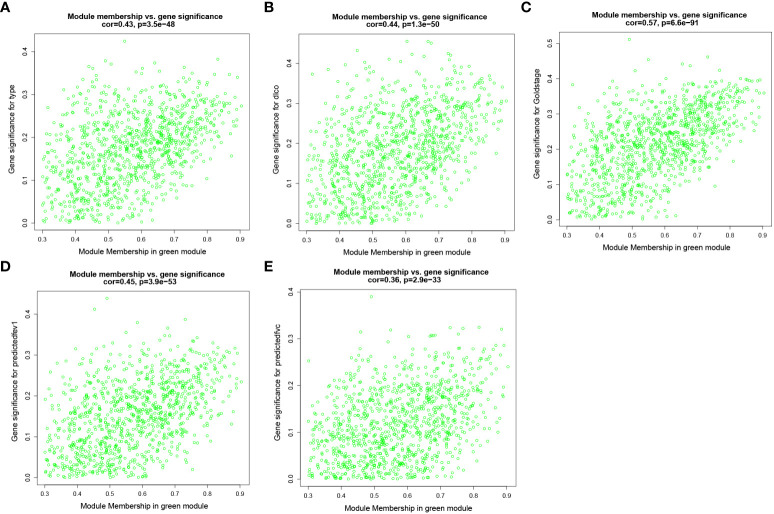
Weighted co-expression network analysis. Scatter plot of genetic significance of emphysema phenotype **(A)**, DLCO **(B)**, GOLD Stage **(C)**, FEV1 post-bronchodilator % predicted **(D)** and FVC post-bronchodilator % predicted **(E)** *vs*. Module Membership in green module.

To identify key genes associated with clinical features of patients with emphysema, a weighted co-expression network based on the GSE47460 dataset was constructed by WGCNA. The clinical information, including age, sex, GOLD Stage, FEV1 post-bronchodilator % predicted, FVC post-bronchodilator % predicted, and DLCO, was included in the analysis. Samples with incomplete information were excluded, and 212 samples with 15,180 genes were selected for further analysis. The samples were clustered by Pearson correlation analysis, and a topological overlap matrix was constructed. Finally, 12 modules were selected based on average hierarchical clustering and dynamic tree clipping (**Figures 6A–C**). The association between modules and clinical traits was measured by the Pearson correlation coefficient using the module eigengene (ME) values and clinical features. As shown in **Figure 6D**, the green and blue modules were closely related to multiple clinical traits, and the correlation coefficients are shown in **Figures 7**, **8**.

### Identification of key genes and experimental validation

**Figure 9:**
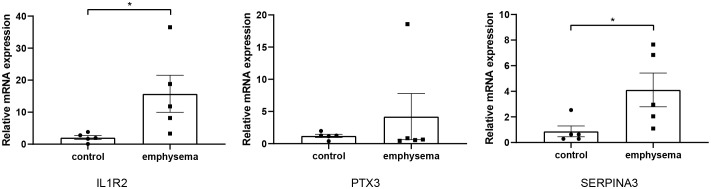
The relative expression level of key genes in emphysematous lung tissues and control tissues. * indicates p < 0.05, respectively.

To obtain DEGs related to immune and clinical characteristics, we intersected the immune-related key genes in the 3.2 section with genes in the green module and the blue module in the 3.3 section. The results showed that the immune key genes had no intersection with genes in the green module. In the blue module, 3 key genes related to immune and clinical features were excavated. To verify key genes more precisely, we detected the mRNA expression levels of three key genes in emphysematous lung tissues and non-emphysematous lung tissues by Rt-qPCR. The results showed that the expression levels of IL1R2 and SERPINA3 in emphysematous lung tissues were significantly higher than those in non-emphysematous lung tissues. In contrast, the expression level of PTX3 did not show a significant difference (p<0.05, **Figure 9**), consistent with their expression trend in the bioinformatics analysis.

### Correlation between expression levels of key genes and tissue-infiltrating immune cells

**Figure 10:**
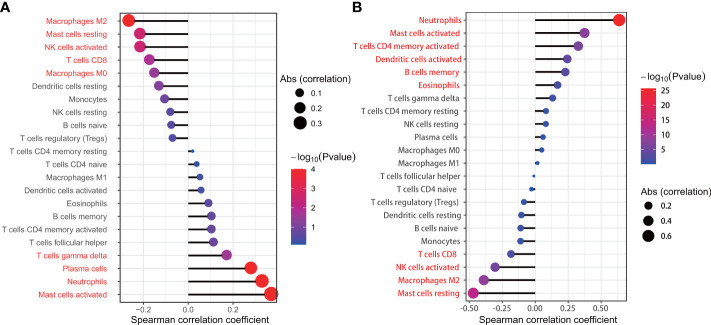
The relationship between SERPINA3 **(A),** IL1R2 **(B)** and the infiltration level of lung tissue-infiltrating immune cells; Statistical significance (P<0.05) in red font.

We used the Wilcoxon signed-rank-sum test to analyze the correlation between immune cell abundance and the expression level of key genes (IL1R2 and SERPINA3) with p<0.05 as the critical value. The results are shown in **Figure 10**, we found that resting mast cells (r=-0.469, p<0.001), macrophages M2 (r=-0.389, p<0.001), activated NK cells (r=-0.304, p<0.001), CD8+ T cells (r=-0.183, p=0.007), eosinophils (r=0.17, p=0.012), memory B cells (r=0.229, p=0.001), activated dendritic cells (r=0.244, p<0.001), activated CD4+ memory T cells (r=0.327, p<0.001), activated mast cells (r=0.375, p<0.001), neutrophils (r=0.637, p<0.001) were significantly associated with IL1R2, while resting mast cells (r=-0.222, p=0.003), macrophages M2 (r=-0.272, p<0.001), activated NK cells (r=-0.221, p=0.003), CD8+ T cells (r=-0.179, p=0.015), macrophages M0 (r=-0.157, p=0.035), T cells gamma delta (r=0.166, p=0.025), plasma cells (r=0.273, p<0.001), activated mast cells (r=0.363, p<0.001), neutrophils (r=0.322, p<0.001) were significantly associated with SERPINA3.

### Construction of emphysema risk prediction model

**Figure 11:**
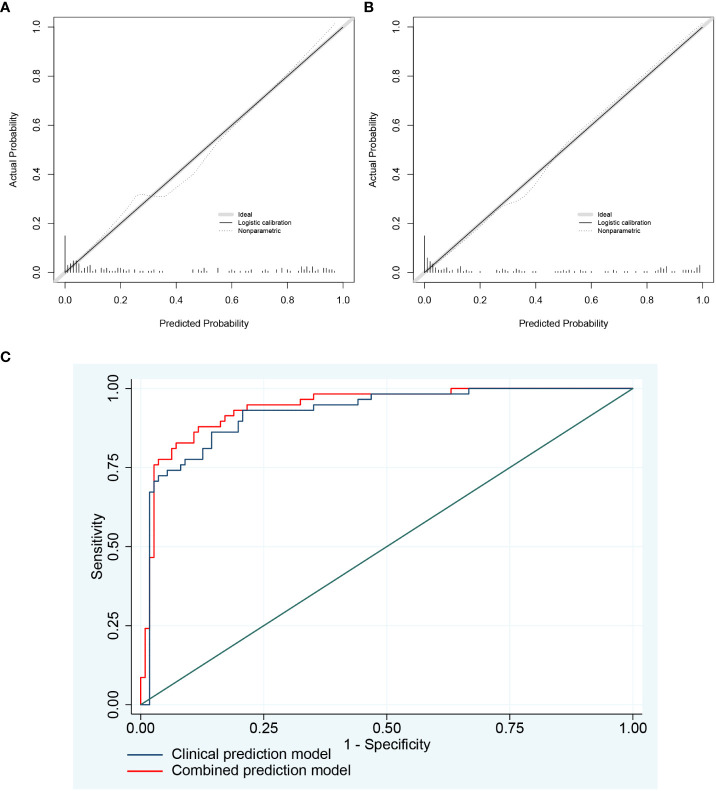
The calibration plots and ROC curves of two prediction models. **(A)** The calibration plots of the clinical prediction model; **(B) **The calibration plots of the combined prediction model. As shown by the nonparametric line, the predicted probability of the combined prediction model is closer to the actual probability compared with the clinical prediction model. **(C)** The ROC curves of two prediction models.

**Figure 12:**
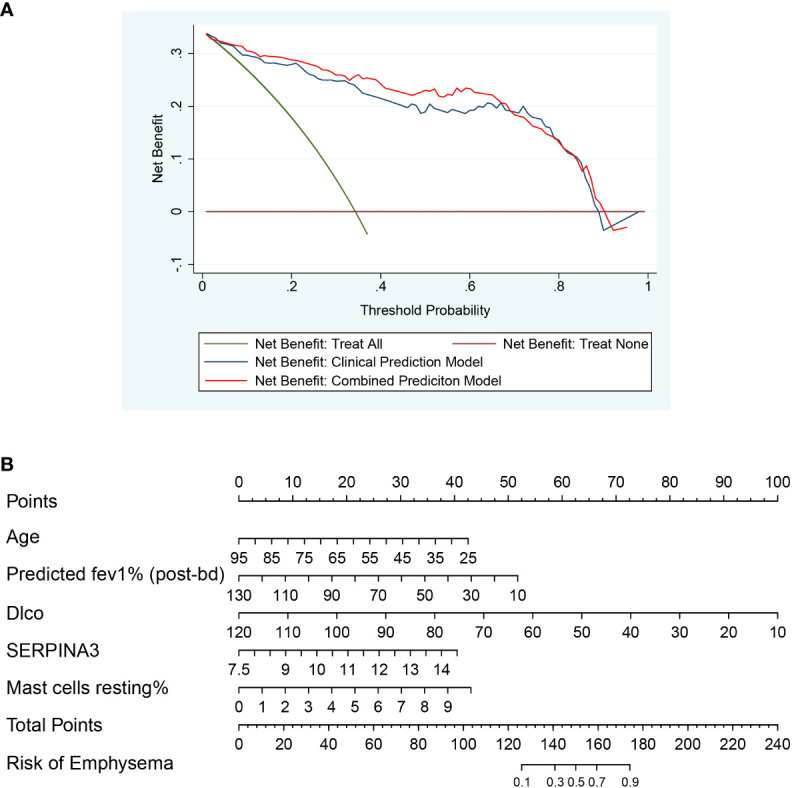
The DCA plot and nomogram. **(A)** The DCA plot of two prediction models, which shows that the combined prediction model has a wider threshold probability and higher net benefit than the clinical prediction model; **(B)** The nomogram of the combined prediction model.

After univariate analysis and multivariate logistic regression analysis, two models were constructed for predicting emphysema risk: the clinical prediction model and the combined prediction model. Age, FEV1 post-bronchodilator % predicted, and DLCO were included in the clinical prediction model, while SERPINA3 and resting mast cells were added in the combined prediction model. The p-values for the clinical and combined predictive models were greater than 0.05 in the H-L test (0.815 and 0.149, respectively), indicating qualified goodness of fit in both models. In addition, the calibration and ROC curves show that the combined prediction model has better calibration and discrimination power (**Figure 11**). The AUC value of the combined prediction model (0.941) was higher than that of the clinical prediction model (0.923). The DCA showed that the combined prediction model had a higher net benefit (**Figure 12A**). A nomogram of the combined prediction model was developed (**Figure 12B**).

### Validation of key genes and tissue-infiltrating immune cells

**Figure 13:**
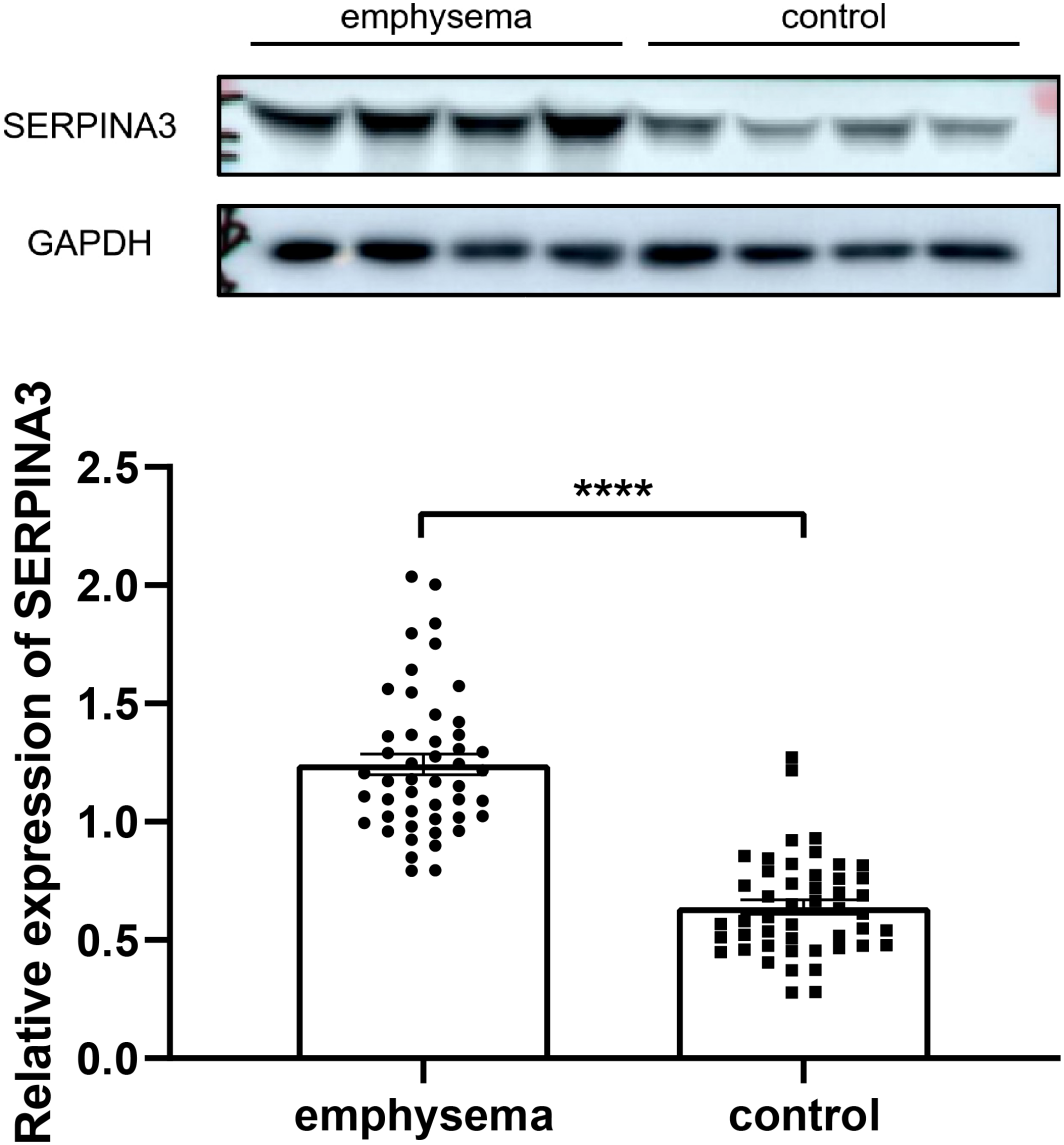
Western blotting results of expression level of SERPINA3 in the lung tissue samples of emphysematous and control groups of COPD. **** indicates p < 0.0001, respectively.

The expression of SERPINA3 was determined in the present study. Each lung tissue sample obtained from a subject was used as an independent sample in a western blot. Western blotting results showing the expression of SERPINA3 in the lung tissue samples of the validation cohort are shown in **Figure 13**. The protein expression of SERPINA3 in the emphysema samples was significantly higher than in the non-emphysema samples.

**Figure 14:**
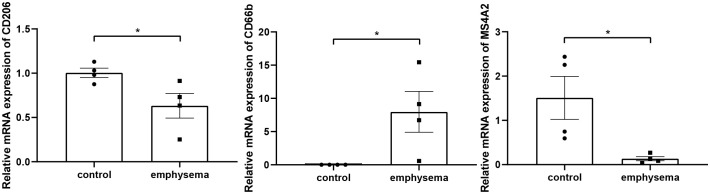
The relative expression level of markers of key tissue-infiltrating immune cells in emphysematous lung tissues and control tissues. * indicates p < 0.05, respectively.

Rt-qPCR analyses were performed to validate the expression levels of CD206, CD66b, and MS4A2 in lung tissues, which are markers of macrophages M2, neutrophils, and resting mast cells. The results showed that the infiltration levels of macrophages M2, neutrophils, and resting mast cells significantly differed between emphysema and non-emphysema phenotypes. The results were consistent with the results obtained by bioinformatics (**Figure 14**).

## Discussion

Since the development of the elastase/anti-elastase theory and subsequent theories about oxidative damage, immune imbalance, and inflammation, the pathogenesis of emphysema has been better understood. In addition to neutrophils and macrophages, many new immune cells, such as T cells follicular helper and T cells gamma delta, have been found to play a role in the progression of emphysema, which makes the landscape of emphysema disease clearer, and also increases our understanding of the importance of immune cells in the occurrence and development of emphysema.

In this study, 6 types of immune cells were found to be significantly associated with emphysema, including neutrophils, T cells follicular helper, resting NK cells, monocytes, M2 macrophages, and resting mast cells. Various types of cells, including B cells memory, Tregs, T cells gamma delta, and macrophages, were found to be associated with clinical features (FEV1 post-bronchodilator % predicted, GOLD Stage, and DLCO). Such diverse immune cells do not act alone in the lung tissue microenvironment but are more inclined to interact with others and synergistically affect the process of emphysema (17, 20). Macrophages and dendritic cells release cytokines such as CXCL8 under the stimulation of smoking, pathogen invasion, and other factors, leading to the recruitment of neutrophils. These pro-inflammatory cells participate in extracellular matrix degradation, mucus secretion, and cell damage by releasing ROS, proteases, some inflammatory factors, and chemokines, promoting the development of emphysema (21, 22). Tregs attenuate the inflammatory response and the resulting lung damage through a contact-dependent mechanism and cytokines such as IL-10, IL-35, and TGF-β, slowing down the aggravation of clinical symptoms (23). The immune cell trends in this study are consistent with the emphysema development process mentioned above.

Some immune cells associated with emphysema or clinical features draw our attention. As a specialized CD4+ T cell subset, follicular helper T (Tfh) cells are critical for forming lymphoid organs. The tertiary lymphoid organs are associated with the severity and tissue damage of emphysema. In 2020, Naessens T et al. demonstrated the existence of Tfh-like cells in the GOLDI/II stage for the first time and found that CD1c+ conventional dendritic cells (cDC) induced naïve CD4+ T-cells into IL-21 and CXCL13 secreting Tfh-like cells by expressing OX40L and the two cells (CD1c+ cDC and Tfh-like cells) co-localized in the tertiary lymphoid organs (24). We found that Tfh cells were significantly elevated in the emphysema group, supporting the abovementioned point.

Another notable immune cell is memory B cells, which were found to be negatively correlated with FEV1 post-bronchodilator % predicted, DLCO, and positively correlated with GOLD Stage in this study (p<0.05). Memory B cells are generated upon the first encounter with a pathogen and can enhance responses to secondary antigen stimulation. A recent study found that the number of IgA+ memory B cells was significantly increased in patients with small airway dysfunction (25). Another study also found an increase in IgA+ B cells in peripheral lung tissue of patients with severe COPD, possibly due to intraluminal sIgA deficiency. Memory B cells may drive inflammation and remodeling by producing antibodies against self-antigens (26).

As a very small subset of T cells, T cells gamma delta is less common in the human immune system with unique T cell receptors (γ-chain and δ-chain) on their surface. Gamma delta T cells were upregulated in smokers’ lung parenchyma compared to non-smokers (27). However, the function of these cells has been unclear and may be related to tissue damage and remodeling (28). In this study, this group of cells was not significantly correlated with emphysema but showed a negative correlation with DLCO and a positive correlation with the GOLD stage. It was recently found that under the induction of IL-1β and IL-23, T cells gamma delta produces IL-17 to promote inflammation (29). Macrophages can secrete IL-1β and IL-23, which in this study was a key immune cell population. In GSE1122, IL-1β and IL-23 were significantly upregulated in the emphysema group, while IL-17 was also upregulated, and the p-value was close to 0.05 (p=0.0547). Therefore, we speculated that T Cells gamma delta also plays a pro-inflammatory role in emphysema in a similar manner, which needs to be further verified.

In this study, neutrophils, macrophages M2, and mast cells resting were significantly correlated with emphysema and clinical features, suggesting that these cells have more significant clinical significance or play a more important role in developing emphysema. It is not surprising that neutrophils were found to be key immune cells. As a classic inflammatory cell, the accumulation of neutrophils in the peri-alveolar tissue is one of the early key events in emphysema and runs through the entire pathogenesis of emphysema. Neutrophils exert pro-inflammatory effects by releasing some cytokines, such as CXCL1 and CXCL8; on the other hand, they cause tissue damage by releasing elastase. The released reactive oxygen species are involved in various processes of emphysema (22). Recent studies have found that neutrophil depletion in allergic airway inflammation increases G-CSF, exacerbating T helper cell type 2 (Th2) inflammation, epithelial remodeling, and airway resistance. This finding suggests that, in addition to pro-inflammatory effects, neutrophils are likely to play an immunomodulatory role (30).

In this study, M2 was found to be a key immune cell which was significantly decreased in the emphysema group. Current studies do not roughly divide macrophages into M1 and M2 subtypes, especially in tumor-related studies. Unfortunately, currently, there is no systematic classification of macrophage subtypes in emphysema or COPD. The single-cell sequencing study by Nassir et al. in patients with critical COVID-19 is of significance, in which monocyte-derived alveolar macrophages were divided into two clusters and one macrophage subtype, labeled CCL3L1 and FCGR3B, was identified to be significantly upregulated in critical COVID-19 (31). Since the classification and function of macrophage subtypes in emphysema are not yet clear, many studies still refer to the classification of M1 and M2 subtypes (32, 33), exerting pro-inflammatory and repairing functions, respectively. The M1 subtype mainly exists in the small airway tissue, while the M2 subtype exists in the luminal area (34). In the present study, M2 macrophages were significantly reduced in the emphysema group, which may be due to the fact that M2 macrophages were damaged in chronic smoke exposure and they were unable to suppress the persistent inflammation leading to the development of emphysema. This is consistent with the findings of Hackett and Takanashi et al., in which the number of IL-10-positive macrophages were reduced in the sputum of COPD patients (35, 36). M2 macrophages can be further divided into 4 subtypes, M2a, M2b, M2c, M2d (37, 38). In this study, CCL-17 was significantly decreased in the emphysema group, whereas IL-13 and IL-4 were elevated. CCL-17 is a cytokine secreted by M2a subtype and can bind to CCR4 recruiting Th2 and Treg cells, which is also one of the ways that M2a subtype exerts anti-inflammatory effect (37). IL-13 and IL-4 can induce M2a polarization (37). The reduction of CCL-17 suggests that the reduction of M2 macrophages in emphysema is mainly dominated by the M2a subtype, and the reduction of the M2a subtype in turn stimulates the increase of IL-13 and IL-4, and also leads to a decrease in anti-inflammatory capacity.

Mast cells are widely involved in inflammation and hypersensitivity reactions *in vivo* by secreting heparin, histamine, and serotonin. The infiltration of mast cells in the airway myometrium is of great significance to the pathogenesis of asthma by affecting airway responsiveness and airway remodeling. In addition, the distribution of mast cell subsets in the lung differs considerably between COPD and healthy individuals, which are associated with pulmonary function changes in patients with COPD. The latest study also found that mast cells stimulate macrophages to release TNF-α by secreting chymase-1 in COPD (39), which further reflects the active interaction of immune cells in emphysema. Andersson et al. found that the infiltration of mast cells was increased in the connective tissue of patients with COPD. At the same time, it was decreased in mucosal tissue, and the infiltration of overall resting mast cells was decreased, which is consistent with the conclusion of this study. Activation and destruction of resting mast cells by smoking and pathogen invasion may be the main reasons for their reduction (40, 41). Activated mast cells were found to be involved in pro-inflammatory and tissue remodeling in emphysema; however, resting mast cells have received less attention from researchers. In the present study, activated mast cells showed an increasing trend in emphysema; however, the difference was not significant (p=0.109), while resting mast cells showed a close association with emphysema and clinical features (FEV1 post-bronchodilator % predicted, DLCO, and GOLD Stage). This finding suggests that resting mast cells may be more responsive to the severity of emphysema.

SERPINA3 was found to be a key immune molecule associated with COPD emphysema through a series of bioinformatics analyses in this study. SERPINA3 is mainly derived from liver and epithelial-derived cells and plays an important role in immunity and acute/chronic inflammatory responses (42, 43). As an acute response protein, its concentration increases with the aggravation of inflammatory responses (44, 45). The level of SERPINA3 can be affected by various immune cells and cytokines. IL-1β and TNF-α can stimulate the expression of SERPINA3 in the U373 cell line *via* the NF-κB pathway (46, 47). Oncostatin M (a member of the IL6 family) induces high expression of SERPINA3 in patients with IBD by phosphorylating STAT1/3 and activating the JAK-STAT pathway important for promoting chronic inflammation (48, 49). SERPINA3 has also been found to be involved in a variety of physiological functions such as complement cascade, apoptosis, wound healing, and extracellular matrix remodeling, but it has been less studied in emphysema phenotypes (50–52). In the present study, we found that SERPINA3 was significantly upregulated in emphysema phenotype, and multiple immune cells (such as neutrophils, macrophages, NK cells, mast cells, and T cells) were associated with its up-regulation. We speculate that under the stimulation of smoke and pathogens, immune cells such as macrophages and neutrophils secrete IL-6, IL-1β, and TNF-α, causing peripheral chronic inflammation, which stimulates the up-regulation of SERPINA3. High levels of SERPINA3 suggest a continuous accumulation of pro-inflammatory factors in the body, which can exacerbate lung tissue damage and cause emphysema. This notion is supported by the findings of Pelin et al. that serum SERPINA3 levels are associated with poor outcomes in COPD, including worsening systemic inflammatory status and increased 10-year mortality (53). Studies have shown that SERPINA3 may be one of the terminal molecules of the inflammatory cascade: soluble IL-6 receptors can restore IL-6-mediated activation of SERPINA3, and various inflammatory factors can affect its expression level (48). The level of SERPINA3 *in vivo* is normally maintained at 0.3–0.6 mg/mL, and it can surge to 4-fold within 8 hours after infection, which can be used for rapid detection of acute inflammation (44). Therefore, we speculate that SERPINA3 can be used as a sensitive indicator for early warning of emphysema, and SERPINA3 was also found to be an independent risk factor for emphysema risk in logistic regression analysis.

A nomogram is a visual form of a multivariate regression model. The influence of each variable on the outcome is represented by a line segment with ticks. The user can get the probability of the outcome by calculating a score based on the value of each variable. The nomogram is intuitive and user-friendly. It was initially used in cancer prediction and is now gradually extended to other diseases. In this study, we sought to use the identified key immune cells and genes to predict emphysema risk. Mast cells resting and SERPINA3 were found to have the predictive value. A combined prediction model and corresponding nomogram were developed that combined “clinical traits + key immune cells + key genes” to predict the risk of emphysema. Compared with the clinical prediction model with only clinical features, the combined prediction model has better discrimination power, calibration power, and a higher net benefit, suggesting that combining immune cells and genes can more accurately predict emphysema risk. The combined prediction model and corresponding nomogram may allow earlier treatment and intervention in patients with emphysema.

The limitation of this study is that the data involved are online data. Due to the scarcity of emphysema-related data, only two eligible online datasets were retrieved. Fortunately, the datasets included the sequencing data of 220 patients, which is a relatively large number in the sequencing analysis and ensures the accuracy and reliability of our analysis results. In addition, only Rt-qPCR verification was performed on the expression level of key genes screened by WGCNA in lung tissue. The expression level of key genes in serum and bronchoalveolar lavage fluid was not verified due to the limitation of clinical sample types. In this study, the CIBERSORT deconvolution method was used to analyze the subtypes and numbers of 22 immune cells in the lungs of 220 patients with COPD. The advantage of this method is that it breaks the dependence of traditional flow cytometry techniques on limited libraries of phenotypic markers and can detect multiple types of immune cells at the same time (19). In addition, although single-cell mRNA sequencing enables unbiased transcriptional analysis of thousands of single cells from single-cell suspensions, analysis of large sample sizes is still difficult to achieve, and sample preparation is limited (54).

In this study, we focused on the emphysema phenotype of COPD, analyzed the immune cells infiltrating the lung tissue, and found the key immune genes associated with emphysema through a series of bioinformatics analyses. A combined prediction model and nomogram were developed to predict emphysema risk better.

## Data availability statement

The datasets presented in this study can be found in online repositories. The names of the repository/repositories and accession number(s) can be found in the article/**Supplementary Material**.

## Ethics statement

The studies involving human participants were reviewed and approved by the Medical Ethics Committee of China Medical University. The patients/participants provided their written informed consent to participate in this study.

## Author contributions

DW and BC participated in the study design and manuscript drafting. SB took part in the acquisition of data. DW and BC partook in the analysis and interpretation of data. LZ contributed to the study supervision. All authors contributed to the article and approved the submitted version.

## Funding

This research was supported by National Natural Science Foundation of China (No. 82170047), and the 345 Talent project of Shengjing Hospital (No. M0423).

## Acknowledgments

Thanks for the samples provided by the Biobank of Shengjing Hospital of China Medical University. Simultaneously, we are very grateful to the patients who agreed to participate in the study.

## Conflict of interest

The authors declare that the research was conducted in the absence of any commercial or financial relationships that could be construed as a potential conflict of interest.

## Publisher’s note

All claims expressed in this article are solely those of the authors and do not necessarily represent those of their affiliated organizations, or those of the publisher, the editors and the reviewers. Any product that may be evaluated in this article, or claim that may be made by its manufacturer, is not guaranteed or endorsed by the publisher.

## References

[1] Organization WH . Chronic obstructive pulmonary disease (Copd) (2022). Available at: https://www.who.int/news-room/fact-sheets/detail/chronic-obstructive-pulmonary-disease-(copd) (Accessed May 22, 2022).

[2] Rennard SI Vestbo J . The many "Small copds": Copd should be an orphan disease. Chest (2008) 134(3):623–7. doi: 10.1378/chest.07-3059 18779197

[3] Halpin DMG Criner GJ Papi A Singh D Anzueto A Martinez FJ . Global initiative for the diagnosis, management, and prevention of chronic obstructive lung disease. the 2020 gold science committee report on covid-19 and chronic obstructive pulmonary disease. Am J Respir Crit Care Med (2021) 203(1):24–36. doi: 10.1164/rccm.202009-3533SO 33146552 PMC7781116

[4] Snider GL Kleinerman J Thurlbeck W and Bengali ZH . The definition of emphysema. Report of a national heart, lung, and blood institute, division of lung diseases workshop. Am Rev Respir Dis (1985) 132(1):182–5. doi: 10.1164/arrd.1985.132.1.182 4014865

[5] O'Donnell DE James MD Milne KM Neder JA . The pathophysiology of dyspnea and exercise intolerance in chronic obstructive pulmonary disease. Clin Chest Med (2019) 40(2):343–66. doi: 10.1016/j.ccm.2019.02.007 31078214

[6] Smith BM Prince MR Hoffman EA Bluemke DA Liu CY Rabinowitz D . Impaired left ventricular filling in copd and emphysema: Is it the heart or the lungs? the multi-ethnic study of atherosclerosis copd study. Chest (2013) 144(4):1143–51. doi: 10.1378/chest.13-0183 PMC3787914 23764937

[7] Sheen S Sun JS Park JH Oh YM Ki SK Kim K . Unique features of non-obstructive emphysema and pure airway obstruction. Int J Tuberc Lung Dis (2014) 18(1):109–16. doi: 10.5588/ijtld.13.0258 24365562

[8] Bai S Ye R Wang C Sun P Zhao L . Comparative analysis of pathophysiological parameters between emphysematous smokers and emphysematous patients with copd. Sci Rep (2020) 10(1):420. doi: 10.1038/s41598-019-57354-2 31942006 PMC6962428

[9] Saetta M Turato G Maestrelli P Mapp CE Fabbri LM . Cellular and structural bases of chronic obstructive pulmonary disease. Am J Respir Crit Care Med (2001) 163(6):1304–9. doi: 10.1164/ajrccm.163.6.2009116 11371392

[10] Porto BN Stein RT . Neutrophil extracellular traps in pulmonary diseases: Too much of a good thing? Front Immunol (2016) 7:311. doi: 10.3389/fimmu.2016.00311 27574522 PMC4983612

[11] D'Anna SE Maniscalco M Cappello F Carone M Motta A Balbi B . Bacterial and viral infections and related inflammatory responses in chronic obstructive pulmonary disease. Ann Med (2021) 53(1):135–50. doi: 10.1080/07853890.2020.1831050 PMC7877965 32997525

[12] Geerdink JX Simons SO Pike R Stauss HJ Heijdra YF Hurst JR . Differences in systemic adaptive immunity contribute to the 'Frequent exacerbator' copd phenotype. Respir Res (2016) 17(1):140. doi: 10.1186/s12931-016-0456-y 27793198 PMC5084432

[13] Robinson AB Stogsdill JA Lewis JB Wood TT Reynolds PR . Rage and tobacco smoke: Insights into modeling chronic obstructive pulmonary disease. Front Physiol (2012) 3:301. doi: 10.3389/fphys.2012.00301 22934052 PMC3429072

[14] Stogsdill MP Stogsdill JA Bodine BG Fredrickson AC Sefcik TL Wood TT . Conditional overexpression of receptors for advanced glycation end-products in the adult murine lung causes airspace enlargement and induces inflammation. Am J Respir Cell Mol Biol (2013) 49(1):128–34. doi: 10.1165/rcmb.2013-0013OC 23526218

[15] Hlapcic I Hulina-Tomaskovic A Somborac-Bacura A Rajkovic MG Dugac AV Popovic-Grle S . Extracellular adenosine triphosphate is associated with airflow limitation severity and symptoms burden in patients with chronic obstructive pulmonary disease. Sci Rep (2019) 9(1):15349. doi: 10.1038/s41598-019-51855-w 31653924 PMC6814706

[16] Schneider JL Rowe JH Garcia-de-Alba C Kim CF Sharpe AH Haigis MC . The aging lung: Physiology, disease, and immunity. Cell (2021) 184(8):1990–2019. doi: 10.1016/j.cell.2021.03.005 33811810 PMC8052295

[17] Caramori G Casolari P Barczyk A Durham AL Di Stefano A Adcock I . Copd immunopathology. Semin Immunopathol (2016) 38(4):497–515. doi: 10.1007/s00281-016-0561-5 27178410 PMC4897000

[18] Loh LC Ong CK Koo HJ Lee SM Lee JS Oh YM . A novel ct-emphysema Index/Fev1 approach of phenotyping copd to predict mortality. Int J Chron Obstruct Pulmon Dis (2018) 13:2543–50. doi: 10.2147/COPD.S165898 PMC6110287 30174423

[19] Newman AM Liu CL Green MR Gentles AJ Feng W Xu Y . Robust enumeration of cell subsets from tissue expression profiles. Nat Methods (2015) 12(5):453–7. doi: 10.1038/nmeth.3337 PMC4739640 25822800

[20] Hogg JC Chu F Utokaparch S Woods R Elliott WM Buzatu L . The nature of small-airway obstruction in chronic obstructive pulmonary disease. N Engl J Med (2004) 350(26):2645–53. doi: 10.1056/NEJMoa032158 15215480

[21] Belchamber KBR Donnelly LE . Targeting defective pulmonary innate immunity - a new therapeutic option? Pharmacol Ther (2020) 209:107500. doi: 10.1016/j.pharmthera.2020.107500 32061706

[22] Wang C Zhou J Wang J Li S Fukunaga A Yodoi J . Progress in the mechanism and targeted drug therapy for copd. Signal Transduct Target Ther (2020) 5(1):248. doi: 10.1038/s41392-020-00345-x 33110061 PMC7588592

[23] Eriksson Strom J Pourazar J Linder R Blomberg A Lindberg A Bucht A . Airway regulatory T cells are decreased in copd with a rapid decline in lung function. Respir Res (2020) 21(1):330. doi: 10.1186/s12931-020-01593-9 33317530 PMC7734742

[24] Naessens T Morias Y Hamrud E Gehrmann U Budida R Mattsson J . Human lung conventional dendritic cells orchestrate lymphoid neogenesis during chronic obstructive pulmonary disease. Am J Respir Crit Care Med (2020) 202(4):535–48. doi: 10.1164/rccm.201906-1123OC PMC7616955 32255375

[25] Habener A Grychtol R Gaedcke S DeLuca D Dittrich AM Happle C . Iga(+) memory b cells are significantly increased in patients with asthma and small airways dysfunction. Eur Respir J (2022) 60(3):2102130. doi: 10.1183/13993003.02130-2021 PMC9630610 35595320

[26] Ladjemi MZ Martin C Lecocq M Detry B Nana FA Moulin C . Increased iga expression in lung lymphoid follicles in severe chronic obstructive pulmonary disease. Am J Respir Crit Care Med (2019) 199(5):592–602. doi: 10.1164/rccm.201802-0352OC 30339768

[27] Majo J Ghezzo H Cosio MG . Lymphocyte population and apoptosis in the lungs of smokers and their relation to emphysema. Eur Respir J (2001) 17(5):946–53. doi: 10.1183/09031936.01.17509460 11488331

[28] Rennard SI . Inflammation and repair processes in chronic obstructive pulmonary disease. Am J Respir Crit Care Med (1999) 160(5 Pt 2):S12–6. doi: 10.1164/ajrccm.160.supplement_1.5 10556162

[29] Jin C Lagoudas GK Zhao C Bullman S Bhutkar A Hu B . Commensal microbiota promote lung cancer development *Via* gammadelta T cells. Cell (2019) 176(5):998–1013 e16. doi: 10.1016/j.cell.2018.12.040 30712876 PMC6691977

[30] Patel DF Peiro T Bruno N Vuononvirta J Akthar S Puttur F . Neutrophils restrain allergic airway inflammation by limiting Ilc2 function and monocyte-dendritic cell antigen presentation. Sci Immunol (2019) 4(41):eaax7006. doi: 10.1126/sciimmunol.aax7006 31704734 PMC7613621

[31] Nassir N Tambi R Bankapur A Al Heialy S Karuvantevida N Khansaheb HH . Single-cell transcriptome identifies Fcgr3b upregulated subtype of alveolar macrophages in patients with critical covid-19. iScience (2021) 24(9):103030. doi: 10.1016/j.isci.2021.103030 34458692 PMC8384759

[32] Le Y Cao W Zhou L Fan X Liu Q Liu F . Infection of mycobacterium tuberculosis promotes both M1/M2 polarization and mmp production in cigarette smoke-exposed macrophages. Front Immunol (2020) 11:1902. doi: 10.3389/fimmu.2020.01902 32973788 PMC7468417

[33] Santana KG Righetti RF Breda CNS Dominguez-Amorocho OA Ramalho T Dantas FEB . Cholesterol-ester transfer protein alters M1 and M2 macrophage polarization and worsens experimental elastase-induced pulmonary emphysema. Front Immunol (2021) 12:684076. doi: 10.3389/fimmu.2021.684076 34367144 PMC8334866

[34] Eapen MS Hansbro PM McAlinden K Kim RY Ward C Hackett TL . Abnormal M1/M2 macrophage phenotype profiles in the small airway wall and lumen in smokers and chronic obstructive pulmonary disease (Copd). Sci Rep (2017) 7(1):13392. doi: 10.1038/s41598-017-13888-x 29042607 PMC5645352

[35] Takanashi S Hasegawa Y Kanehira Y Yamamoto K Fujimoto K Satoh K . Interleukin-10 level in sputum is reduced in bronchial asthma, copd and in smokers. Eur Respir J (1999) 14(2):309–14. doi: 10.1034/j.1399-3003.1999.14b12.x 10515406

[36] Hackett TL Holloway R Holgate ST Warner JA . Dynamics of pro-inflammatory and anti-inflammatory cytokine release during acute inflammation in chronic obstructive pulmonary disease: An *ex vivo* study. Respir Res (2008) 9:47. doi: 10.1186/1465-9921-9-47 18510721 PMC2435536

[37] Mantovani A Sica A Sozzani S Allavena P Vecchi A Locati M . The chemokine system in diverse forms of macrophage activation and polarization. Trends Immunol (2004) 25(12):677–86. doi: 10.1016/j.it.2004.09.015 15530839

[38] Grinberg S Hasko G Wu D Leibovich SJ . Suppression of Plcbeta2 by endotoxin plays a role in the adenosine a(2a) receptor-mediated switch of macrophages from an inflammatory to an angiogenic phenotype. Am J Pathol (2009) 175(6):2439–53. doi: 10.2353/ajpath.2009.090290 PMC2789640 19850892

[39] Liu G Jarnicki AG Paudel KR Lu W Wadhwa R Philp AM . Adverse roles of mast cell chymase-1 in chronic obstructive pulmonary disease. Eur Respir J (2022) 60(3):2101431. doi: 10.1183/13993003.01431-2021 35777766

[40] Virk H Arthur G Bradding P . Mast cells and their activation in lung disease. Transl Res (2016) 174:60–76. doi: 10.1016/j.trsl.2016.01.005 26845625

[41] Mortaz E Folkerts G Redegeld F . Mast cells and copd. Pulm Pharmacol Ther (2011) 24(4):367–72. doi: 10.1016/j.pupt.2011.03.007 21463700

[42] Heit C Jackson BC McAndrews M Wright MW Thompson DC Silverman GA . Update of the human and mouse serpin gene superfamily. Hum Genomics (2013) 7:22. doi: 10.1186/1479-7364-7-22 24172014 PMC3880077

[43] Hwang SR Steineckert B Kohn A Palkovits M Hook VY . Molecular studies define the primary structure of Alpha1-antichymotrypsin (Act) protease inhibitor in alzheimer's disease brains. comparison of act in hippocampus and liver. J Biol Chem (1999) 274(3):1821–7. doi: 10.1074/jbc.274.3.1821 9880565

[44] Aronsen KF Ekelund G Kindmark CO Laurell CB . Sequential changes of plasma proteins after surgical trauma. Scand J Clin Lab Invest Suppl (1972) 124:127–36. doi: 10.3109/00365517209102760 5041008

[45] Janciauskiene S . Conformational properties of serine proteinase inhibitors (Serpins) confer multiple pathophysiological roles. Biochim Biophys Acta (2001) 1535(3):221–35. doi: 10.1016/s0925-4439(01)00025-4 11278163

[46] Kordula T Bugno M Rydel RE Travis J . Mechanism of interleukin-1- and tumor necrosis factor alpha-dependent regulation of the alpha 1-antichymotrypsin gene in human astrocytes. J Neurosci (2000) 20(20):7510–6. doi: 10.1523/JNEUROSCI.20-20-07510.2000 PMC6772857 11027208

[47] Lieb K Fiebich BL Schaller H Berger M Bauer J . Interleukin-1 beta and tumor necrosis factor-alpha induce expression of alpha 1-antichymotrypsin in human astrocytoma cells by activation of nuclear factor-kappa b. J Neurochem (1996) 67(5):2039–44. doi: 10.1046/j.1471-4159.1996.67052039.x 8863511

[48] Kordula T Rydel RE Brigham EF Horn F Heinrich PC Travis J . Oncostatin m and the interleukin-6 and soluble interleukin-6 receptor complex regulate Alpha1-antichymotrypsin expression in human cortical astrocytes. J Biol Chem (1998) 273(7):4112–8. doi: 10.1074/jbc.273.7.4112 9461605

[49] West NR Hegazy AN Owens BMJ Bullers SJ Linggi B Buonocore S . Oncostatin m drives intestinal inflammation and predicts response to tumor necrosis factor-neutralizing therapy in patients with inflammatory bowel disease. Nat Med (2017) 23(5):579–89. doi: 10.1038/nm.4307 PMC5420447 28368383

[50] Hsu I Parkinson LG Shen Y Toro A Brown T Zhao H . Serpina3n accelerates tissue repair in a diabetic mouse model of delayed wound healing. Cell Death Dis (2014) 5:e1458. doi: 10.1038/cddis.2014.423 25299783 PMC4237249

[51] Reiss MJ Han YP Garner WL . Alpha1-antichymotrypsin activity correlates with and may modulate matrix metalloproteinase-9 in human acute wounds. Wound Repair Regener (2009) 17(3):418–26. doi: 10.1111/j.1524-475X.2009.00476.x 19660051

[52] Hoffmann DC Textoris C Oehme F Klaassen T Goppelt A Romer A . Pivotal role for Alpha1-antichymotrypsin in skin repair. J Biol Chem (2011) 286(33):28889–901. doi: 10.1074/jbc.M111.249979 PMC3190696 21693707

[53] Takei N Suzuki M Makita H Konno S Shimizu K Kimura H . Serum alpha-1 antitrypsin levels and the clinical course of chronic obstructive pulmonary disease. Int J Chron Obstruct Pulmon Dis (2019) 14:2885–93. doi: 10.2147/COPD.S225365 PMC6911326 31849461

[54] See P Lum J Chen J Ginhoux F . A single-cell sequencing guide for immunologists. Front Immunol (2018) 9:2425. doi: 10.3389/fimmu.2018.02425 30405621 PMC6205970

